# Epidemiological impact and cost-effectiveness of universal meningitis b vaccination among college students prior to college entry

**DOI:** 10.1371/journal.pone.0239926

**Published:** 2020-10-09

**Authors:** Grace S. Chung, David W. Hutton

**Affiliations:** Department of Health Management and Policy, University of Michigan, Ann Arbor, Michigan, United States of America; Icahn School of Medicine at Mount Sinai, UNITED STATES

## Abstract

**Objectives:**

University students are at significantly higher risk of serogroup B meningococcal (MenB) infection, which can result in debilitating sequelae and excessive healthcare usage. This study aimed to elucidate the impact of universal pre-enrollment vaccination on MenB outbreak probability and the cost-effectiveness in outbreak-only scenarios.

**Methods:**

We developed an infectious disease transmission model to determine the number of outbreaks averted under universal vaccination and a Markov model to simulate the costs accrued and QALYs lost associated with infection. The analysis was done on a hypothetical population of 40,000 college students over a four-year time frame. We used the outputs of these two models to calculate the incremental cost-effectiveness ratio (ICER) of universal MenB vaccination from a societal perspective.

**Results:**

We find that the vaccination strategy was estimated to reduce MenB incidence by 63% and outbreak frequency rate by 90%. Under base case assumptions, the ICER of universal vaccination was $748,129 per QALY and in outbreak-only scenarios, it was cost-saving.

**Conclusions:**

Universal vaccination is not cost-effective at the current low MenB incidence levels and vaccine price in the U.S., but it is cost-saving if outbreak is imminent.

## Introduction

Undergraduate students are a large, important population that present a unique challenge to the public health apparatus in our country. They are at an increased risk of infectious disease outbreaks due to multiple factors including proximity of living quarters, common dining facilities, and shared food and drinks [1, 2]. Of these infectious diseases, arguably the most severe is meningococcal meningitis, caused by the bacteria *Neisseria meningitidis*. Though rare, this infection is serious and can be fatal in 10% of cases, even with treatment within 24 hours. Of those who survive, 10–20% will suffer serious long-term disabilities, including hearing loss, kidney damage, amputations, skin grafts, or neurological disability [1, 2]. Due to the severity of disease, a series of vaccinations is recommended for students matriculating into U.S. colleges to prevent outbreaks that can spread rapidly among dormitory-dwelling students.

Specifically, the US Centers for Disease Control and Prevention (CDC) immunization schedule recommends routine vaccination for all 11 to 12 year-olds, with a booster dose at age 16, against the most common subgroups of *N*. *meningitidis* in the United States: serogroups A, C, Y, and W. Over the past 20 years, the overall incidence of meningococcal ACYW disease in the US has declined substantially, leaving serogroup B as the leading cause of meningococcal outbreaks [2]. Serogroup B meningococcal disease (MenB) is responsible for 50% of all meningococcal disease cases among 17–22 year olds [3, 4] and 100% of meningococcal disease outbreaks on college campuses in the U.S. [5] Since 2009, there have been seven outbreaks of MenB on college campuses, resulting in 43 cases and 3 deaths [2]. Yet, undergraduate students are not explicitly listed in the current recommendation for meningitis B vaccination from the CDC^2^, and 83% of 17-year-olds have not received at least one dose of the MenB vaccine [4]. This raises the question of whether meningitis B vaccination should be required for all entering college students, who are more than five times more likely to acquire MenB than non-college students [6].

Though a recently published cost-utility analysis (CUA) by Leeds et al. [7] reported that universal vaccination at college entry is not cost-effective, it did not estimate the number and severity of outbreaks reduced through vaccination or the cost-effectiveness of the intervention in outbreak-only scenarios. Other cost-effectiveness studies have been limited to pediatric populations in the UK [8–11], Germany [12], and non-comparable lower-income settings, such as the meningitis belt in Africa [13–15]. Therefore, we aimed to use an infectious disease dynamic transmission model to assess the impact of universal pre-enrollment vaccination on the number of cases and outbreaks as well as maximum outbreak size in a hypothetical college population. We then conducted a CUA to determine whether it would be cost effective for MenB vaccination to be mandated for matriculating students, as compared to these schools continuing the status quo practice of deploying vaccination campaigns against this disease only in direct response to outbreaks, and a secondary analysis in outbreak-only scenarios. Elucidating the costs and health implications of meningitis B vaccination is important to prioritize various immunization activities for college students.

## Methods

We developed an infectious disease transmission model, a Markov model and an economic model and used them in conjunction with each other to examine the impact of routine administration of two doses of the MenB-4C (Bexsero) or MenB-FHbp (Trumenba) vaccines prior to matriculation in a hypothetical population of 40,000 college students from a societal perspective. We used a four-year time frame, as college students are typically in school for four years. Immunization schedules were based on MenB vaccine immunogenicity and efficacy studies [16, 17].

**Fig 1:**
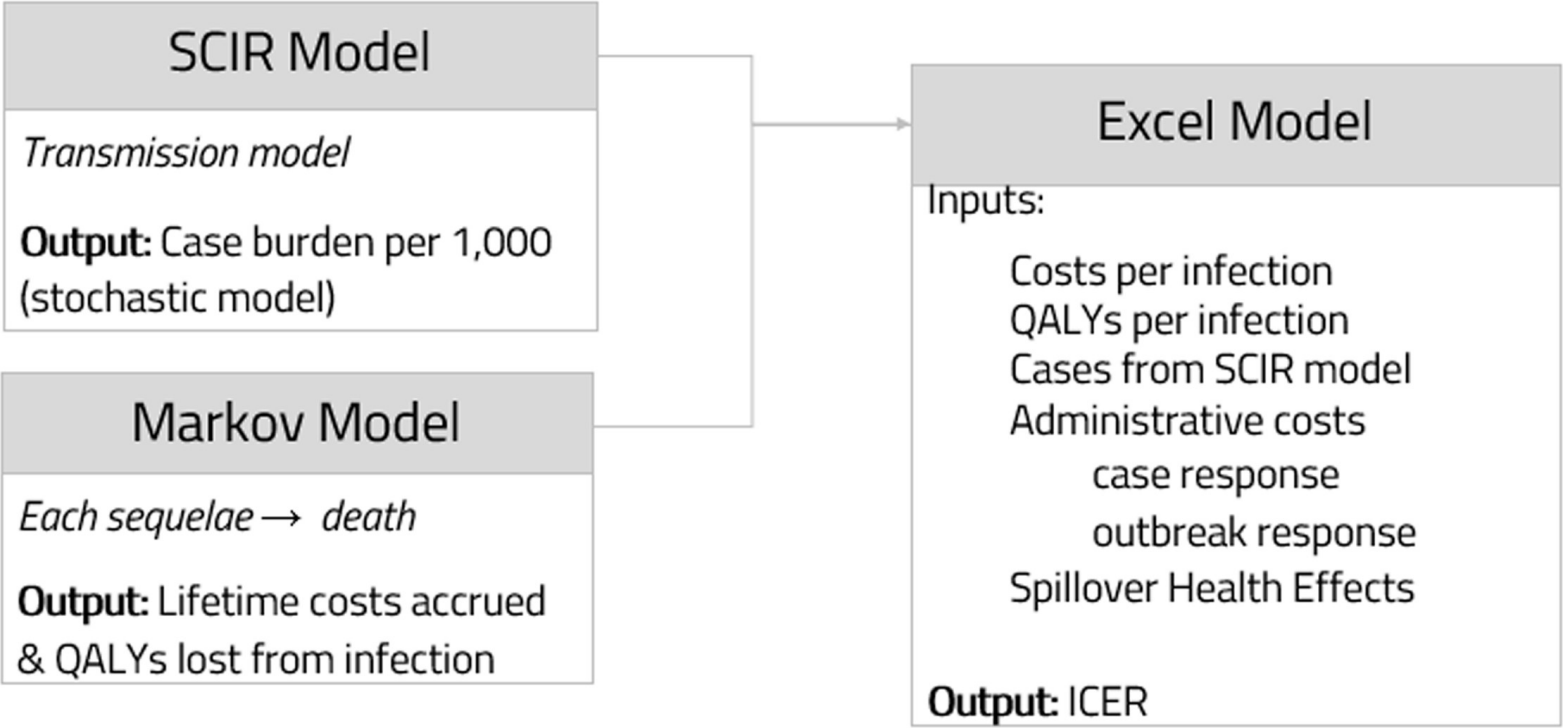
Overall modeling approach.

We used a dynamic transmission model to estimate the number of cases and outbreaks as well as maximum outbreak size in both the base case and 90% vaccination coverage scenarios and a Markov model to simulate the costs and QALYs associated with MenB infection. We used the outputs of these two models as inputs into our economic model in Excel, which added societal spillover effects and overhead outbreak response costs, in order to determine the overall ICER as well as the ICER in outbreak-only scenarios (Fig 1). We first evaluated the ‘status quo’ practice of deploying vaccination campaigns against MenB *only in direct response* to outbreaks to create a baseline for comparison to the universal vaccination strategy. Second, the costs and QALYs associated with a pre-matriculation vaccination program were calculated.

**Fig 2:**
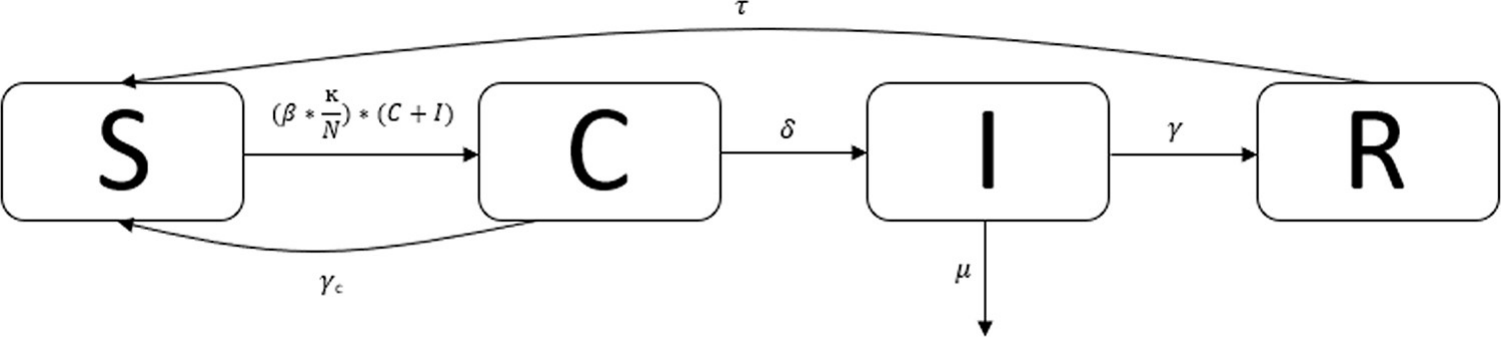
SCIR dynamic infectious disease model.

A stochastic compartmental SCIR model was used to calculate disease dynamics of meningitis on a hypothetical college campus (Fig 2). The compartments represent susceptible individuals (S), carriers (C), infected individuals (I), and recovered individuals (R). Stochasticity was approximated using the tau-leaping method and assuming Poisson distributions. Additionally, N is the total population size, *β* represents the infection transmission parameter, ĸ represents the contact rate between students, *δ* represents the risk of disease given carriage, *γ* represents the reciprocal of the average duration of recovery, *μ* represents the disease-associated mortality rate, and *τ* is the reciprocal of the average duration of immunity. Further, individuals can remain in a carriage state and never proceed to infection and return to the susceptible class, represented by *γ_c_*.

Our parameters were drawn from the literature and are shown in Table 1. We assumed that students enter college at the age of 18 and that carriers are half as infectious as cases. The SCIR model is represented by the following set of differential equations:

$$\frac{dS}{dt}=-\beta *ĸ/N\;*\;(C+I)\;*\;S+\gamma_{c}\;*\;C+\tau \;*\;R$$

$$\frac{dC}{dt}=\beta \;*\;ĸ/N\;*\;(C+I)\;*\;S‐\gamma_{c}\;*\;C‐\delta \;*\;C$$

$$\frac{dI}{dt}=\delta *C-\mu *I-\gamma *I$$

$$\frac{dR}{dt}=\gamma *I‐\tau \;*\;R$$

We calibrated the model to the MenB incidence estimate of 0.23 per 100,000 persons for the college-going, 18-24-year-old U.S. population in 2016 from the CDC^3^ and the maximum outbreak size of 9 from Soeters et al. [18] by tweaking the initial number of cases and carriers. The behavior of the model remained relatively stable over the 4-year simulation period, with the incidence rate ranging from 0.04 to 0.08 per 100,000 people per calendar quarter (S1 Fig).

**Table 1:** Parameters.

Parameter		Value (Range)	Distribution	Reference
*β* (transmission rate)		0.00085 (0.00075–0.00095)	Nml	Poore & Bauch (2015) [19]
ĸ (contact rate)		10.86 (4.19–17.53)	Nml	Mossong et al. (2008) [20]
*δ* (rate of progression to disease given carriage)		0.000870 (0.000812–0.000933)	Nml	Trotter et al. (2006) [21]
*μ* (disease mortality, per 100,000 people)		0.000315 (0.000137–0.000493)	Nml	Pace & Pollard (2012) [22]
*γ_c_* (rate of recovery from carriage)		0.00658 (0.00365–0.00951)	Nml	Trotter et al. (2006) [21]
*γ*(rate of recovery from infection)		0.152† (0.083–0.383)	Nml	Institute of Medicine (US) Committee (2000) [23]
				Batista et al. (2017) [24]
τ (rate of immunity loss)		0.00137 (0.000913–0.00274)	Nml	Poore & Bauch (2015) [19]
*q* (vaccine efficacy), %		73 (57–87)	Nml	Vogel et al. (2013) [25]
Adverse Reactions		0‖		
	***Meningococcal Disease Sequelae Probabilities***			
Skin scarring, %		7.6 (0–19)	Nml, trunc	Shepard et al. (2005) [26]
Single amputation, %		1.9 (0.5–10)	Nml, trunc	Shepard et al. (2005) [26]
Multiple amputations, %		1.2 (0.02–6)	Nml, trunc	Shepard et al. (2005) [26]
Hearing loss, %		6.4 (2–20)	Nml, trunc	Shepard et al. (2005) [26]
Neurologic disability, %		2.1 (0.02–11)	Nml, trunc	Shepard et al. (2005) [26]
Case fatality rate, %		9.3 (3.1–13.7)	Nml	Ortega-Sanchez et al. (2008) [27]
	***Sequelae-specific health-utility weights***			
Acute Disease		0.0033 (0–0.0066)	Nml	Institute of Medicine (US) Committee (2000) [23]
Skin scarring		1 (0.8–1)	Nml, trunc	Ortega-Sanchez et al. (2008) [27]
Single amputation		0.70 (0.31–0.80)	Nml	Ortega-Sanchez et al. (2008) [27]
Multiple amputation		0.61 (0.31–0.71)	Nml	Ortega-Sanchez et al. (2008) [27]
Hearing loss		0.72 (0.64–0.82)	Nml	Ortega-Sanchez et al. (2008) [27]
Long-term neurologic disability		0.06 (0–0.39)	Nml, trunc	Ortega-Sanchez et al. (2008) [27]
	***Costs associated with our models and health states***			
Acute Disease	*Initial hospitalization*, *$*	52,868 (45,997–64,264)	Nml	Davis et al. (2011) [28]
Lifetime Costs of Meningococcal Sequelae	*Skin scarring*, *$*	7,378 (3,689–11,067)	Nml	Shepard et al. (2005) [26]
	*Single amputation*, *$*	215,356 (107,677–323,034)	Nml	Shepard et al. (2005) [26]
	*Multiple amputations*, *$*	258,427 (129,213–387,639)	Nml	Shepard et al. (2005) [26]
	*Hearing loss*, *$*	88,879 (33,912–143,846)	Nml	Shepard et al. (2005) [26]
				CDC (2004) [29]
	*Neurologic disability*, *$*	3,241,896 (1,195,167–4,257,677)	Nml	Shepard et al. (2005) [26]
Vaccination Costs	Cost of vaccination per person, $	310.27 (50.00–500.00)	Nml	CDC Vaccine Price List (2020) [30]
	Vaccine wastage, %	10 (0–25)	Nml	Ortega-Sanchez et al. (2008) [27]
	Cost of vaccine administration per person, $	28.88* (24.06–43.30)	Nml	CMS (2020) [31]
Outbreak Response Costs	Costs by University and other entities, $	14,500,000 (13,200,000–15,800,000)	Nml	La et al. (2018) [32]
Productivity (friction cost approach)	Productivity costs of skin scarring, amputation, neurological deficit, or hearing loss	0	Nml	Leeds et al. (2019) [7]
	Productivity costs of early death	9,174 (8,916–9,432)	Nml	Leeds et al. (2019) [7]
Discount Rate, %		3 (1–5)	Nml	Sanders et al. (2016) [33]

Trunc indicates truncated; nml, normal.

† *γ* was determined by taking the reciprocal of the average of the duration of recovery without complications and the duration

of recovery with serious long-lasting sequelae.

‖ We assumed that the incidence of adverse reactions (1/900,000) from vaccination is negligible.

*Vaccine administration costs were assumed to be $12.03 per dose in a mass vaccination and either $14.44 for administration

during an existing visit to a clinician in a physician office setting or $21.65 for administration during an extra physician office

setting visit based on Medicare payment rates [31].

The vaccination proportion, *p*, was included not as a separate compartment, but rather as a proportion of the initial conditions of susceptibility (i.e. the number of susceptibles). This proportion was multiplied by vaccine efficacy, *q*. As such, (1—p*q) *S determined the initial number of susceptibles in the simulations with vaccination.

We created a Markov model of infected vs. uninfected arms to project the average costs accrued and quality-adjusted life-years (QALYs) lost per infected individual over a lifetime. In the case of infection, we modeled the progression from recovered without sequelae to death and from each of the different sequelae upon recovery from meningococcal disease (skin scarring, single amputation, multiple amputation, hearing loss, and neurologic disability) to death based on the probabilities of being in each health state (S2 Fig). We assumed that neurologic disability is the only sequelae that increases risk of mortality over a lifetime and used cerebral palsy mortality as a proxy for mortality from neurologic disability, as cerebral palsy can cause seizures which will more appropriately capture the variability of possible outcomes from neurologic disability [34]. We treated each of the sequelae as a mutually exclusive event, and assumed that those who died from infection died after getting medical care such that all decedents had medical care costs. In the case of no infection, the possible health states were alive or dead, and we used age-group-specific US Census estimates of average life expectancy in 2000 [35] to obtain the mortality probabilities for all health states other than neurologic disability.

We evaluated three primary outcomes assuming an individual develops the disease, as well as their associated costs and QALYs. Given the fact that the majority of individuals will not be affected by meningitis, we characterized the costs associated with the disease and QALYs *lost* given cases of disease rather than including QALYs of those who are never infected. Thus, we only evaluated outcomes associated with case status:

1. Survival without sequelae (R1)
2. Survival with long-term sequelae (R2) (skin scarring, single amputation, multiple amputation, hearing loss, and neurologic disability)
3. Death (for which we calculated QALYs lost given the difference in the age at death and the age-adjusted life expectancy)

We used a threshold of two or more cases predicted in a given SCIR simulation to trigger an outbreak and ‘outbreak prevention’ measures at the University. If an outbreak was detected, then in addition to our costs associated with the prior three outcomes, the overhead costs of outbreak response and management by the CDC and local health authorities (Epidemic Intelligence Service resources, personnel, ambulances, on-site vaccination administration, etc.) were included in the economic model. For the outbreak response costs, we scaled up the outbreak cost from La et al. [32] to match the size of our hypothetical population compared with the much smaller Providence College. In accordance with the timeline of mass vaccination at Providence College, we assumed that once an outbreak is detected, the vaccination campaign starts immediately.

Additionally, QALY spillover into the student population due to the fear and anxiety about MenB was accounted for through extrapolation from previous work assessing health spillover in caretakers for infected individuals, and adjusted for impact on the student body [36]. We used this spillover effect (a loss of 1/100th of a QALY per person) for half the campus, which was the assumed proportion significantly affected by being in close proximity to an infected individual.

Previous literature elucidates the impact on quality of life and costs from extrapolation from similar diseases and from meningococcal illness specifically. The transition probabilities, health-utilities and costs for each sequela can be found in Table 1. We incorporated additional costs of lost productivity attributable to premature death from meningococcal illness, assessed using a friction costing method. For the vaccine cost, we used an average of the costs of two doses of Bexsero ($341.50) and Trumenba ($279.04) [30].

All costs were inflated to 2018 US dollars using the Consumer Price Index inflation calculator [37]. Based on recommendations of the US Panel on Cost Effectiveness in Health and Medicine, all future costs and benefits were discounted at a 3% annual rate [38].

We conducted a two-way sensitivity analysis of the vaccination population coverage (ranging from 80–99%) and vaccine efficacy (ranging from 57–87%) on the incidence rate and outbreak frequency rate. We ran one-way sensitivity analyses of all parameters in the SCIR model, Markov model, and economic model, varying each parameter within the defined value ranges, to assess the influence of the range of plausible estimates for these parameters on the ICER.

A probabilistic sensitivity analysis varied all parameters simultaneously using a Monte Carlo simulation of 1,000 iterations. Distributions for parameter inputs are described in Table 1. We also examined how the ICER changes with the probability of outbreak. Threshold analyses were conducted to determine the outbreak frequency rate that would make vaccination cost-effective as well as the value for vaccine cost that would lead to a change in the base case results, under a willingness-to-pay (WTP) threshold of $100,000 per QALY.

The Markov model was created using TreeAge Pro 2020 R 2.0 (TreeAge Software, Williamstown, Massachusetts, USA). The SCIR model was created and analyses were performed in R version 3.5.0. The economic model was developed using Microsoft Excel version 19.11 and sensitivity analyses were performed in R and Excel.

## Results

Table 2 shows the results of the simulation model. As expected, MenB infection is rare. The incidence rate of meningitis in the absence of vaccination is 0.23 per 100,000 people per year. The incidence rate and maximum outbreak size in the base case scenario closely match calibration targets (S1 Table). With 90% vaccination (and 73% vaccine efficacy), it is reduced to 0.084 per 100,000 people per year. In addition to the reduction in cases as measured by incidence, there were substantially fewer outbreaks (63 vs. 6) over 1,000 4-year simulations in the base case vs. vaccination scenario, yielding respective outbreak frequency rates of 0.039 and 0.004 per 100,000 people per year. The maximum outbreak size was reduced from 8 to 2.

**Table 2:** Outputs from SCIR model with and without vaccination.

	Base Case (No Vaccination)	90% Vaccination (with 73% Vaccine Efficacy)
Total # of Cases*	362	135
Total # of Outbreaks*	63	6
**Max Outbreak Size**	8	2
**Incidence Rate (per 100,000 people per year)**	0.226	0.084
**Outbreak Frequency Rate (per 100,000 people per year)**	0.039	0.004

* Number of cases and outbreaks are out of 1,000 simulations.

Table 3 shows the incidence rate and outbreak frequency rate for varying levels of vaccination population coverage and efficacy. With 99% vaccination coverage and 87% vaccine efficacy, the MenB incidence rate is reduced to 0.08 per 100,000 people per year and the outbreak frequency rate is reduced to 0.001 per 100,000 people per year. The results of the sensitivity analyses do not show evidence of herd immunity.

**Table 3:** Variable vaccine efficacy and vaccination percent along with the incidence rate, outbreak frequency rate, and maximum outbreak size per 1,000 4-year simulations.

Vaccination %	Efficacy	Susceptible Proportion	Incidence Rate (per 100,000 people per year)	Outbreak Frequency Rate (per 100,000 people per year)	Max Outbreak Size
N/A	N/A	100.0%	0.226	0.039	8
80%	57%	54.4%	0.099	0.008	4
80%	73%	41.6%	0.098	0.005	3
80%	87%	30.4%	0.096	0.003	2
85%	57%	51.5%	0.116	0.007	3
85%	73%	37.9%	0.103	0.006	3
85%	87%	26.0%	0.087	0.003	2
90%	57%	48.7%	0.110	0.007	3
90%	73%	34.3%	0.084	0.004	2
90%	87%	21.7%	0.081	0.001	2
95%	57%	45.8%	0.101	0.005	3
95%	73%	30.6%	0.108	0.003	3
95%	87%	17.3%	0.090	0.001	2
99%	57%	43.6%	0.106	0.004	2
99%	73%	27.7%	0.088	0.004	2
99%	87%	13.9%	0.080	0.001	2

Covering 90% of the population would add almost $13.5 million in net costs. The ICER of MenB vaccination is about $750,000 per QALY, which is not cost effective by any standard WTP threshold (Table 4).

**Table 4:** Lifetime costs and QALYs of 90% meningitis B vaccination intervention vs. status quo across 1,000 simulation runs.

	Vaccination Costs ($)	Meningitis Costs ($)	Total Costs ($)	QALYslost	Incremental Costs	Incremental QALYs	ICER ($ per QALY)
**Status quo**	913,500	49,461	962,961	21.22			
**90% Vaccination**	13,517,340	18,445	13,535,785	4.41	12,572,824	16.81	748,129

However, if we instead calculate the cost of the status quo outbreak response only based on vaccination in simulations where there was an outbreak, we get a very different answer, where vaccinating the entire campus becomes a cost-saving intervention (Table 5).

**Table 5:** Lifetime costs and QALYs of 90% meningitis B vaccination intervention vs. status quo across 1,000 simulation runs with recorded outbreaks.

	Vaccination Costs ($)	Meningitis Costs ($)	Total Costs ($)	QALYs lost	ICER ($ per QALY)
**Status quo**	14,500,000	358,996	14,858,996	262.57	
**90% Vaccination**	13,517,340	18,445	13,535,785	4.41	Cost-saving

One-way sensitivity analyses of all parameters used in the SCIR model, Markov model, and economic model were conducted. We created a tornado diagram showing the ten most influential parameters (S3 Fig). The cost-effectiveness of meningitis B vaccination was most sensitive to the contact rate, followed by rate of recovery from carriage. In a one-way sensitivity analysis of the contact rate, the ICER ranged from $168,088 to $8,690,626. Varying the rate of recovery from carriage led the ICER to range from $160,782 to $2,416,911. But, in the end, varying one parameter at a time did not result in pre-outbreak meningitis B vaccination being cost-effective at a WTP threshold of $100,000 per QALY.

**Fig 3:**
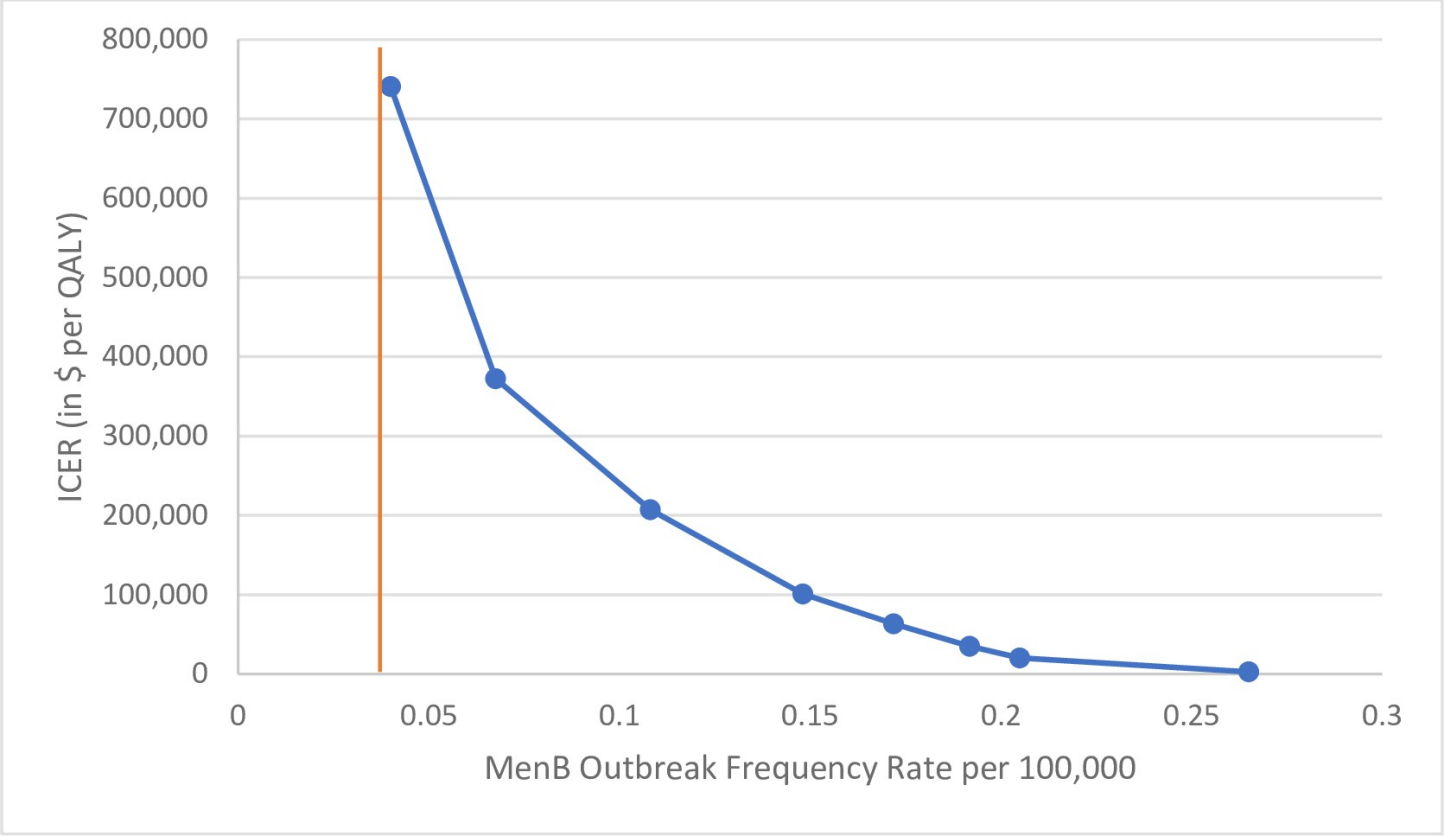
One-way sensitivity analysis of MenB outbreak frequency rate.

We also performed a one-way sensitivity analysis of MenB outbreak frequency rate on the ICER associated with universal vaccination (Fig 3). We found that universal vaccination is cost-effective at the $100,000 WTP threshold at an outbreak frequency rate of about 0.15 per 100,000 per year (and a corresponding incidence rate of 1.6 per 100,000 per year). In addition, threshold analysis showed that the ICER meets the $100,000 WTP threshold at a vaccine cost of $35.21 for two doses.

**Fig 4:**
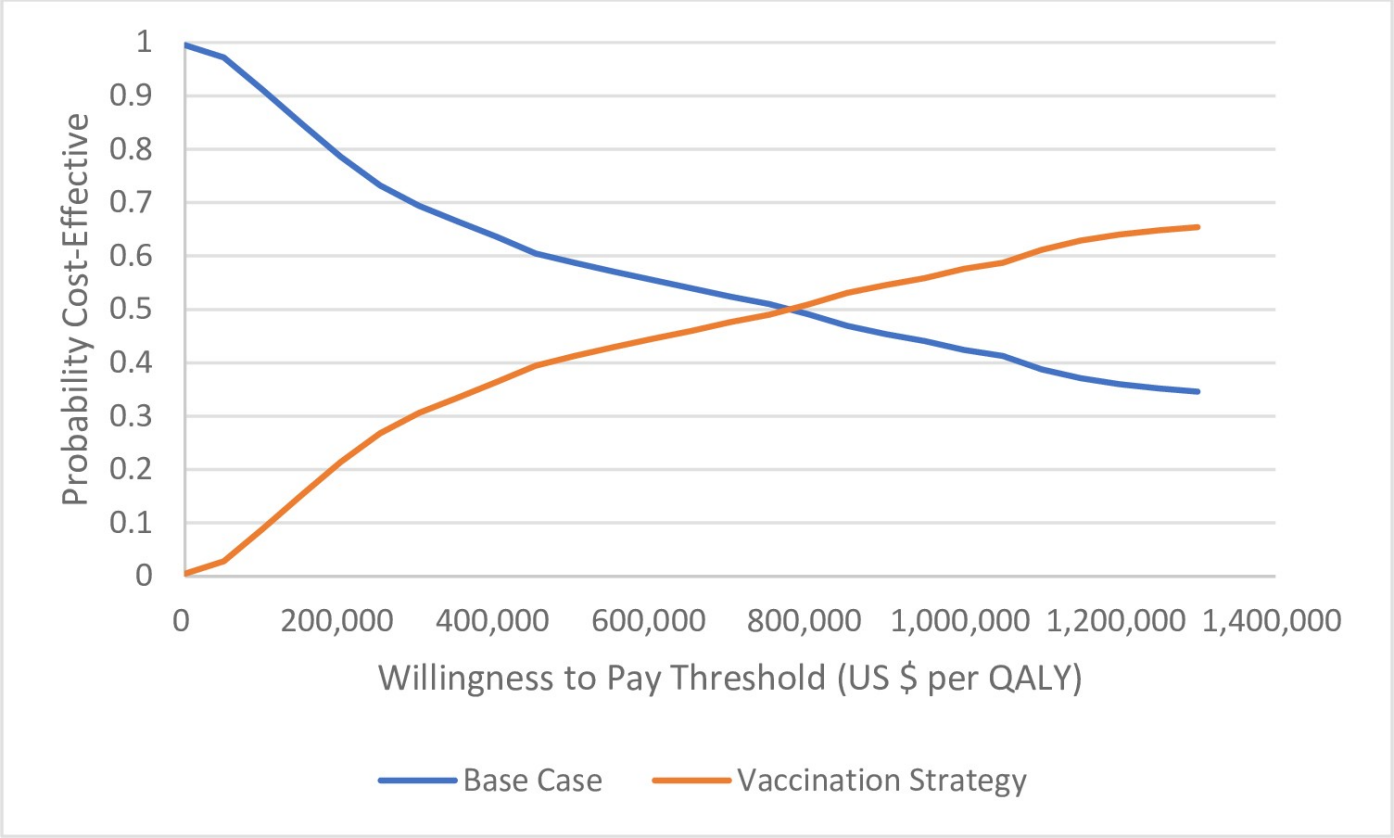
Cost-effectiveness acceptability curves representing the probability that the vaccination strategy is cost-effective for a given maximum willingness-to-pay threshold per QALY gained.

The probabilistic sensitivity analysis varied all parameters simultaneously. S2 Table shows the average costs, QALYs, incremental costs, incremental QALYs, and ICER and 95% uncertainty intervals resulting from the PSA. With a WTP threshold of $100,000 per QALY, universal pre-matriculation vaccination was cost-effective in 8.9% of simulations in the base case scenario. The intervention was more likely to be cost-effective than not at a WTP threshold of about $750,000 (Fig 4).

## Discussion

We find that in the no vaccination scenario, the incidence rate of MenB infection is 0.23 in a hypothetical university population of 40,000 students. This matches the CDC incidence estimate of 0.23 per 100,000 persons for the college-going, 18-24-year-old U.S. population in 2016 [3], which assumes 36.2% of 18–24 year olds attending college. Vaccinating 90% of the population with a vaccine efficacy of 73% was estimated to provide a 63% reduction in MenB incidence and a 90% reduction in outbreak frequency rate. It also reduced the severity of outbreaks.

While we did not find that universally recommending MenB vaccination was cost effective, the secondary analysis performed on outbreak-only scenarios showed cost-savings. If an outbreak could be anticipated, vaccinating is not only life-saving, but cost-saving. In order to inform policy decisions, further research on what certain harbingers of such an outbreak might be is warranted to identify situations which fall under the ‘imminent outbreak prevented’ category. This might include finding subgroups at higher risk within the undergraduate population such as frequent study-abroad travelers, school athletes, and those who are immunosuppressed.

Given that universal vaccination is very expensive in the base case (with a low risk of outbreak), but cost-saving if you know an outbreak is going to happen, we looked at varying the probability of outbreak. The outbreak frequency rate that would make vaccination cost-effective is about 0.15 per 100,000 people per year. Universal vaccination should be reconsidered if there is an observed increase in the frequency of these outbreaks. A threshold analysis also found the intervention cost-effective (at a WTP threshold of $100,000) when the vaccine is $35.21, underscoring the implications of reduced vaccine cost in health policy, though this would be a significant reduction in the cost of the vaccine from its current cost of $279.04-$341.50 for two doses.

Our study does have some limitations. To begin, the analysis does not incorporate the QALY detriment to family members of those students who were sickened or died on campus, which may have underestimated the disease burden and costs associated with an outbreak. Especially if an outbreak is ongoing on campus prior to an academic break, such as Christmas or Thanksgiving, there would be the potential for students either carrying or latently infected with the disease to return home and infect their families. The resulting additional, larger pool of susceptible families might provide a significant source of secondary infections and change the resulting ICER. Further, the cost of outbreak response is hard to ascertain. The costs that we utilized in this model were primarily taken from a recent study, which estimated the cost of outbreak response at a much smaller university (Providence College). While we felt that it was appropriate to scale up the costs to match our population size, this may be an imperfect estimate and adds potential error. In addition, the cost associated with the emotional and psychological stress and fear about an outbreak is difficult to quantify. Not including these costs—be they incurred from psychological counseling or days of school missed–may have significantly underestimated the burden of disease.

## Conclusion

Universal pre-enrollment vaccination against MenB has the potential to reduce the disease burden among college students. Given how rare this disease is, our full-campus vaccination intervention is too expensive to compensate for infrequent, dangerous outbreaks. Thus, without identification of higher-risk groups with a greater likelihood of outbreak, it is hard to justify universal MenB vaccination prior to college. Perhaps smaller-scale, targeted vaccination programs could be both cost-effective and life-saving to select college students across the country.

## Supporting information

S1 Fig Behavior of SCIR model over 4-year simulation period, by calendar quarter. (DOCX)

S2 Fig Markov model. (DOCX)

S3 Fig Sensitivity of the incremental cost-effectiveness ratio for meningitis B vaccination to changes in all parameters. (DOCX)

S1 Table Base case outputs from SCIR model and calibration targets. (DOCX)

S2 Table Average costs, QALYs and ICER resulting from probabilistic sensitivity analysis. (DOCX)
