# Supplementary figures and images for: Epidemiological impact and cost-effectiveness of universal meningitis b vaccination among college students prior to college entry
Source: PLoS One. 2020 Oct 9;15(10):e0239926. doi: 10.1371/journal.pone.0239926 (PMC7546456; doi:10.1371/journal.pone.0239926)

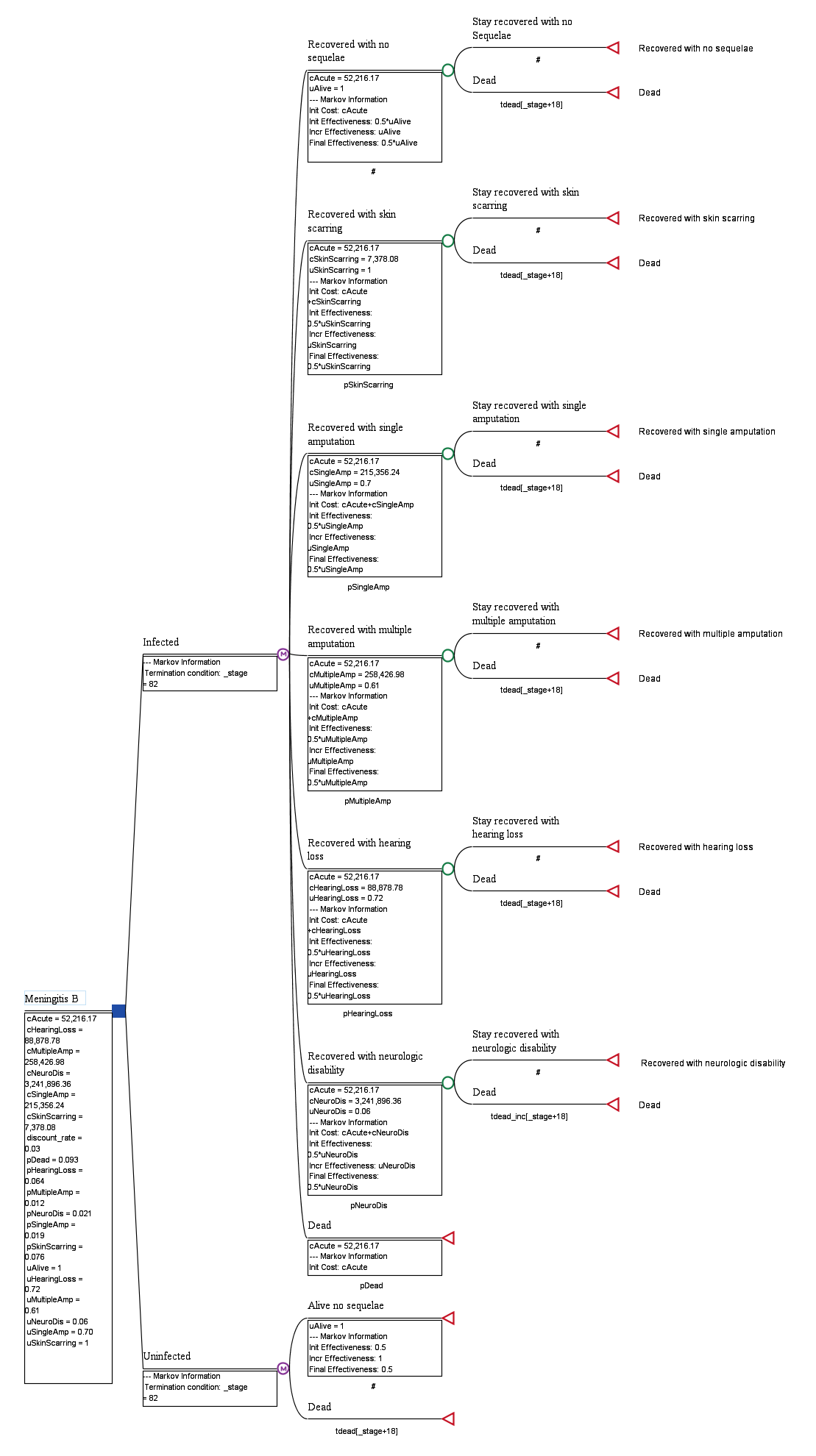

Supplement: S2 Fig — (DOCX) [file pone.0239926.s002.docx]
