# Supplementary material for: Epidemiological impact and cost-effectiveness of universal meningitis b vaccination among college students prior to college entry
Source: PLoS One. 2020 Oct 9;15(10):e0239926. doi: 10.1371/journal.pone.0239926 (PMC7546456; doi:10.1371/journal.pone.0239926)
Supplement: S1 Table — (DOCX) [file pone.0239926.s004.docx]

|  | **Base Case from Model**  **(No Vaccination)** | **Target base case estimates from the CDC [3] and Soeters et al. [40]** |
| --- | --- | --- |
| **Total # of Cases*** | 362 |  |
| **Total # of Outbreaks*** | 63 |  |
| **Max Outbreak Size** | 8 | 9 [40] |
| **Incidence Rate**  **(per 100,000 people per year)** | 0.226 | 0.23 [3] |
| **Outbreak Frequency Rate (per year)†** | 0.016 |  |

* Number of cases and outbreaks are out of 1,000 simulations.

† The outbreak frequency rate is per "representative school of 40,000 students".
