# Supplementary material for: Epidemiological impact and cost-effectiveness of universal meningitis b vaccination among college students prior to college entry
Source: PLoS One. 2020 Oct 9;15(10):e0239926. doi: 10.1371/journal.pone.0239926 (PMC7546456; doi:10.1371/journal.pone.0239926)
Supplement: S2 Table — (DOCX) [file pone.0239926.s005.docx]

|  | **Vaccination Costs ($)** | **Meningitis Costs ($)** | **Total Costs ($)** | **QALYs lost** | **Incremental Costs** | **Incremental QALYs** | **ICER ($ per QALY)** |
| --- | --- | --- | --- | --- | --- | --- | --- |
|  | **Mean (95% uncertainty interval)** | **Mean (95% uncertainty interval)** | **Mean (95% uncertainty interval)** | **Mean (95% uncertainty interval)** | **Mean (95% uncertainty interval)** | **Mean (95% uncertainty interval)** | **Mean (95% uncertainty interval)** |
| **Status quo** | 1,328,704 (1,252,782, 1,404,625) | 91,890 (85,122, 98,658) | 1,420,594 (1,339,350, 1,501,837) | 33.67  (35.62,  31.72) |  |  |  |
| **90% Vaccination** | 13,566,824 (13,275,390, 13,858,258) | 20,939 (20,169, 21,710) | 13,587,764 (13,296,316, 13,879,211) | 3.93  (4.02,  3.84) | 12,167,170 (11,956,965, 12,377,374) | 29.74 (27.88, 31.60) | 409,109 (378,357, 443,968) |
