# Supplementary material for: Epidemiological impact and cost-effectiveness of universal meningitis b vaccination among college students prior to college entry
Source: PLoS One. 2020 Oct 9;15(10):e0239926. doi: 10.1371/journal.pone.0239926 (PMC7546456; doi:10.1371/journal.pone.0239926)
Supplement: Supplementary file 4 [file pone.0239926.s001.docx]

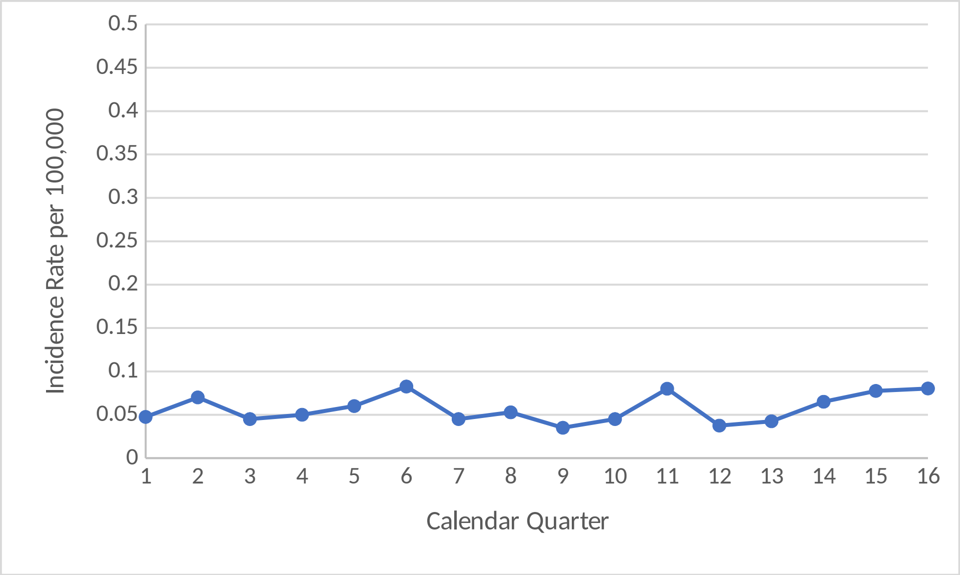

Incidence Rate per 100,000
0.5
0.45
0.4
0.35
0.3
0.25
0.2
0.15
0.1
0.05
0
1 2 3 4 5 6 7 8 9 10 11 12 13 14 15 16
Calendar Quarter
