# Supplementary material for: Epidemiological impact and cost-effectiveness of universal meningitis b vaccination among college students prior to college entry
Source: PLoS One. 2020 Oct 9;15(10):e0239926. doi: 10.1371/journal.pone.0239926 (PMC7546456; doi:10.1371/journal.pone.0239926)
Supplement: Supplementary file 5 [file pone.0239926.s003.docx]

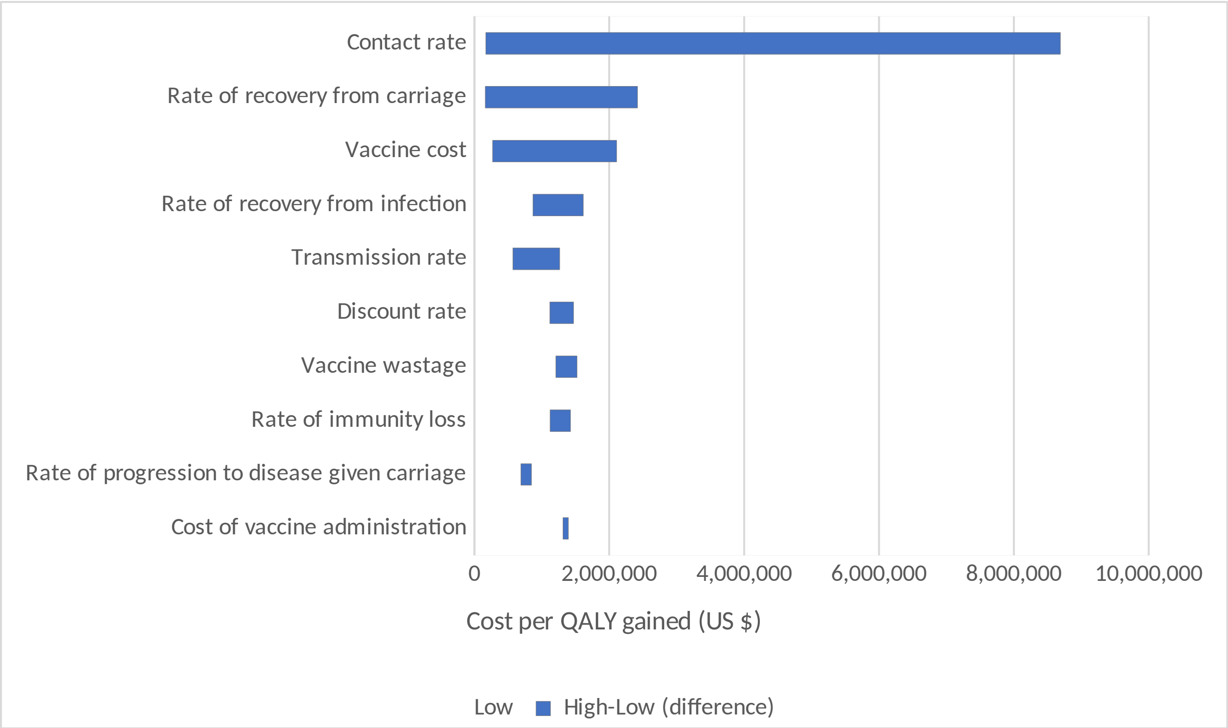
Contact rate
Rate of recovery from carriage
Vaccine cost
Rate of recovery from infection
Transmission rate
Discount rate
Vaccine wastage
Rate of immunity loss
Rate of progression to disease given carriage
Cost of vaccine administration
0
2,000,000
4,000,000
6,000,000
8,000,000
10,000,000
Cost per QALY gained (US $)
Low
High-Low (difference)
